# Statement on advancing the assessment of chemical mixtures and their risks for human health and the environment

**DOI:** 10.1016/j.envint.2019.105267

**Published:** 2019-11-06

**Authors:** Elina Drakvik, Rolf Altenburger, Yasunobu Aoki, Thomas Backhaus, Tina Bahadori, Robert Barouki, Werner Brack, Mark T.D. Cronin, Barbara Demeneix, Susanne Hougaard Bennekou, Jacob van Klaveren, Carsten Kneuer, Marike Kolossa-Gehring, Erik Lebret, Leo Posthuma, Lena Reiber, Cynthia Rider, Joëlle Rüegg, Giuseppe Testa, Bart van der Burg, Hilko van der Voet, A. Michael Warhurst, Bob van de Water, Kunihiko Yamazaki, Mattias Öberg, Åke Bergman

**Affiliations:** a Karolinska Institutet, Institute of Environmental Medicine, Nobels väg 13, SE-171 77 Stockholm, Sweden; b Stockholm University, ACES, SE-106 91 Stockholm, Sweden; c Helmholtz Centre for Environmental Research UFZ, Permoserstr. 15, 04318 Leipzig Germany; d National Institute for Environmental Studies, 16-2 Onogawa, Tsukuba, Ibaraki 305-8506, Japan; e University of Gothenburg Department of Biological and Environmental Sciences, Box 461, SE-405 30 Gothenburg Sweden; f US Environmental Protection Agency, 1200 Pennsylvania Ave, NW, MC 8201R, Washington, DC 20460, USA; g Université de Paris, Inserm Unit 1124, 45 rue des Saints Pères, 75006 Paris, France; h RWTH Aachen University Institute for Environmental Research, ABBt-aachen Biology, Worringerweg 1, 52074 Aachen, Germany; i Liverpool John Moores University, School of Pharmacy and Biomolecular Sciences, Byrom Street, Liverpool L3 3AF, UK; j Muséum National d'Histoire Naturelle (MNHN) UMR 7221 (CNRS/MNHN), 7 rue Cuvier, 75005 Paris, France; k Danish Technical University, FOOD, Kemitorvet 201, 2800 Kgs. Lyngby, Denmark; l National Institute for Public Health and the Environment (RIVM), P.O. Box 1, 3720 BA Bilthoven, the Netherlands; m German Federal Institute for Risk Assessment, Pesticide Safety, German Federal Institute for Risk Assessment, Max-Dohrn-Str. 8-10, 10589 Berlin, Germany; n German Environment Agency (UBA), Corrensplatz 1, 14195 Berlin, Germany; o Institute of Risk Assessment Sciences – IRAS, Utrecht University, Yalelaan 2, 3584 CM Utrecht, the Netherlands; p Radboud University, Department of Environmental Science, Institute for Water and Wetland Research, Nijmegen, the Netherlands; q National Toxicology Program, National Institute of Environmental Health Sciences, 111 TW Alexander Drive, PO Box 12233, MD:K2-12, Research Triangle Park, NC 27709, USA; r Uppsala University, Department of Organismal Biology, Norbyvägen 18A, SE-752 36 Uppsala, Sweden; s University of Milan, Department of Oncology, Via S. Sofia, 9/1, 20122 Milan, Italy; t IEO European Institute of Oncology, Via Adamello 16, 20139 Milan, Italy; u BioDetection Systems, Science Park 406, 1098XH Amsterdam, the Netherlands; v Wageningen University & Research, Droevendaalsesteeg 1, 6708PB Wageningen, the Netherlands; w CHEM Trust, 34b York Way, London N1 9AB, UK; x Leiden Academic Centre for Drug Research, Leiden University, P.O. Box 9502, 2300 RA Leiden, the Netherlands; y Ministry of the Environment, Japan, 1-2-2 Kasumigaseki, Chiyoda-ku, Tokyo 100-8975, Japan; z Örebro University, Department of Science and Technology, SE-701 82 Örebro, Sweden; aa State Key Laboratory of Pollution Control and Resource Reuse, College of Environmental Science and Engineering, Tongji University, Shanghai 200092, China

**Keywords:** Chemical mixtures, Environmental chemicals, Combined exposure, Mixture risk assessment, Risk management

## Abstract

The number of anthropogenic chemicals, manufactured, by-products, metabolites and abiotically formed transformation products, counts to hundreds of thousands, at present. Thus, humans and wildlife are exposed to complex mixtures, never one chemical at a time and rarely with only one dominating effect. Hence there is an urgent need to develop strategies on how exposure to multiple hazardous chemicals and the combination of their effects can be assessed. A workshop, “Advancing the Assessment of Chemical Mixtures and their Risks for Human Health and the Environment” was organized in May 2018 together with Joint Research Center in Ispra, EU-funded research projects and Commission Services and relevant EU agencies. This forum for researchers and policy-makers was created to discuss and identify gaps in risk assessment and governance of chemical mixtures as well as to discuss state of the art science and future research needs. Based on the presentations and discussions at this workshop we want to bring forward the following Key Messages:

• We are at a turning point: multiple exposures and their combined effects require better management to protect public health and the environment from hazardous chemical mixtures.

• Regulatory initiatives should be launched to investigate the opportunities for all relevant regulatory frameworks to include prospective mixture risk assessment and consider combined exposures to (real-life) chemical mixtures to humans and wildlife, across sectors.

• Precautionary approaches and intermediate measures (e.g. Mixture Assessment Factor) can already be applied, although, definitive mixture risk assessments cannot be routinely conducted due to significant knowledge and data gaps.

• A European strategy needs to be set, through stakeholder engagement, for the governance of combined exposure to multiple chemicals and mixtures. The strategy would include research aimed at scientific advancement in mechanistic understanding and modelling techniques, as well as research to address regulatory and policy needs. Without such a clear strategy, specific objectives and common priorities, research, and policies to address mixtures will likely remain scattered and insufficient.

## 1. Introduction

Humans, wildlife and domestic animals are exposed to a large number of different mixtures of anthropogenic chemicals via air, water, food, consumer products, materials and goods. In addition, new chemicals and new applications of existing chemicals are continuously introduced to the market. Pharmaceuticals, drugs, tobacco and occupational exposures add to the number and potential combinations of chemical mixtures for humans. However, the current regulatory practice (Clahsen et al., 2019; Lebret, 2015) is largely based on considering single chemical substances. The combined exposure to multiple chemicals raises concerns about the effects on health and the environment, as the failure to account for the effects of combined exposures could lead to underestimation of risk (Kortenkamp and Faust, 2018).

Scientific progress has been achieved in recent years towards improving our understanding of mixture effects and developing new models to assess risks from combined exposures to multiple chemicals (Bopp et al., 2019). Substantial progress in conceptual approaches to address mixture risks has been achieved and useful methods for default solutions are already available (More et al., 2019). Although evidence of exposure and adverse effects are abundant, there is a significant need to develop methods and search for advanced solutions on how we establish causality between exposure and effects. Also, given an almost infinite number of real-life mixtures will make investigations of all of them unworkable. Simplifications must be done to promote predictions of exposure and effect assessments, which means that approaches for more holistic mixture risk assessments (MRAs) remain a research challenge.

Further, progress has been achieved in improving our understanding of the governance of uncertain, complex and ambiguous risk problems and so-called “wicked problems” that span across various sectors, jurisdictions and agencies (Allen, 2013). Mixture risk assessment certainly qualifies as such a category of risk problems. Such problems do not only require the input of natural and life science experts, but also input from political science, economics, law and from broad stakeholder dialogues (Health Council of the Netherlands, 2008; Petersen and van Asselt, 2008; Renn, 2008; Renn and Graham, 2005).

Many challenges and gaps are yet impeding the progress. Data gaps on the various uses of the large number of chemicals on the market remain, thus hindering more inclusive assessment based on real-life exposures to coincidental mixtures (Bopp et al., 2018). It is inevitable that more work is required to better understand and manage co-exposure to multiple chemicals, both with regards to intentional mixtures such as pesticide formulations and cosmetic products, and unintentional mixtures in our indoor and outdoor environments, combined with the pool of chemical exposures stemming from e.g. pharmaceuticals, medical implants, recreational drugs, tobacco and other lifestyle related exposures.

Against this background, a joint Workshop “Advancing the Assessment of Chemical Mixtures and their Risks for Human Health and the Environment” was held 29–30 May 2018 at Joint Research Centre in Ispra, Italy, co-organised with several EU-funded research projects: EDC-MixRisk, EuroMix, HBM4EU, SOLUTIONS, and EU-ToxRisk. The purpose of this workshop was to create a joint forum for researchers and policy-makers to discuss and identify gaps in risk assessment and governance of chemical mixtures as well as to discuss state of the art research and future research needs. The workshop brought together around 60 experts, with representatives from the EU-funded research projects, Commission services and EU agencies. Participants discussed ways forward for advancing progress in the chemical mixture issue, concerning both human health and the environment.

This paper was prepared in the context of the workshop. The workshop plenary and group discussions form the basis of the present document as well as the additional comments received via a survey among participants after the workshop. The key issues and recommended actions in terms of policy, data and scientific challenges for addressing chemical mixture risks and combined exposures are presented below.

To clarify the terminology used herein, the combined exposure to multiple chemicals is defined as exposure to multiple chemicals via single or multiple sources and/or pathways, whereas aggregate exposure refers to exposure to the same chemical from multiple sources and/or by multiple pathways according to the WHO/IPCS framework (Meek et al., 2011).

The statements and proposals in this paper represent solely the views of the workshop participants and not necessarily the views or official policies of their organizations.

## 2. Current situation and future needs

### 2.1. Policy needs and options

#### 2.1.1. Strengthen the legal basis for mixture risk management and assessment by clear legal mandates

Our current regulatory systems are not designed for coping with co-exposures to multiple chemicals of different application areas presently regulated through different regulatory jurisdictions, e.g. biocides, cosmetic ingredients and additives in material and products. The risk assessment and management of chemicals focuses on single substances while mixtures are only partly covered by the current regulatory frameworks (Kienzler et al., 2014, 2016). The frameworks that only look at compounds from a particular use or application class, e.g. pesticides or biocides, are necessary, but seem insufficient in the light of increasing evidence underlining the importance of considering co- exposures against multiple chemicals beyond application class in order to more adequately estimate risks they pose (Demeneix and Slama, 2019; Evans et al., 2016).

Many workshop participants suggested that a more comprehensive legal basis would help bridge not only regulatory gaps, but also contribute significantly to method development and data provision. The strengthening of the legal basis can be achieved e.g. by establishing clear legal mandates for mixture risk assessment within all sectors of EU chemicals, environmental and waste related legislation. Currently, the EU pesticides legislation (Regulation (EC) No 1107/2009 and Regulation (EC) 396/2005 on pesticide residues) and the biocides legislation (Regulation (EU) No 528/2012) have specific provisions regarding the risk assessment of combined effects (Kienzler et al., 2014). If legal provisions would be added to other chemical related legislations, progress in this area would be possible. Furthermore, many participants highlighted that based on the experiences of the US and the EU regulatory systems (Rotter et al., 2018), it seems that significant progress towards the effective inclusion of mixture risk assessments into regulatory decision cannot be achieved without clear legal mandates requiring competent authorities to develop and implement relevant methodologies.

- “Review” of all pieces of EU chemical related legislation, regardless of their origin, be it environmental, waste, occupation, food, diet, consumer products or pharmaceuticals, to enable fuller, more inclusive mixture impact assessments
  A comprehensive review would enable a topical scanning of the existing horizontal and sector-specific legislations and their provisions and guidance on considering mixture risks in prospective chemical risk assessment as well as in, ex-ante impact assessment. Moreover, issues for cross-compliance between different sectors of regulation, e.g. emission-based and quality-based standard setting could be identified.
- Establish, in stakeholder dialogue, a “protection goal” for human health with regards to chemical exposures which is embedded in regulations
  The Water Framework Directive 2000/60/EC stipulates good chemical and ecological status for European water bodies. Good chemical status requires that concentration of pollutants do not exceed environmental quality standards set at the EU level. A similar type of protection goal could be set for good chemical status in the human population. The goal could be limited to specific domains, e.g. non-voluntary exposures such as environmental, occupational and dietary exposures in the first instance. The status of the protection goal could be assessed via human biomonitoring, which can be used to identify exposure trends in the population or after a set period of time to evaluate effectiveness of policy decisions and measures.

#### 2.1.2. Strengthen coordination across regulatory bodies and sectors

Mistakes due to lack of coordination and communication, as previously made in history, should be avoided. For example, several chemicals originally used as pesticides have been banned but continued to be produced for other applications, such as flame retardants. Where procedures for mixture risk assessments have already been drafted or implemented under different pieces of EU law, they are fragmented and not fully consistent in terminology and assessment rules. Different rules and data requirements apply for the chemicals used for different purposes and/or which are present in different environmental media. As more frameworks, both horizontal (REACH) and sector-specific, should include mixture risk assessment in legislation, it is important that in this process, mixture risk management and risk assessment are approached by the different sectors simultaneously in a coordinated manner. At least, most relevant sources of exposure should be considered to begin with a limited but relevant scope. These include environmental, occupational and dietary exposures, as such involuntary exposures should not impede or limit the elected use of e.g. pharmaceuticals.

It is suggested that mixture risk assessment could drive the harmonisation of chemical and environmental legislation as the current substance-by-substance assessment has its limitations. Harmonised methodologies would ensure consistency, simplify interpretation and help building confidence and support for integration into the regulatory decision-making. To this end, novel cross-cutting initiatives and in-stitutional arrangements were brought up in the discussions in order to enable dialogue between scientific experts and policy makers. “Policy labs” or “pilot platforms” could be initiated to find and introduce suitable procedures and processes for cross-sector coordination and harmonization in various regulatory bodies. Although methodologies developed with data rich sectors of regulation, such as for medicinal products or pesticides, certainly have the potential to drive development of methods, there may be limitations to the application of these within other areas where less data is available.

- Case study to assess mixtures of substances covered by many sectors
  It was proposed as a concrete step forward to initiate a case study across regulatory areas to work together with the European Commission and Agencies for a practical learning experience. A case study would help concretizing the processes and drive the development of cross-sector coordination forward. An evaluation of fungicides was suggested, where similarly acting compounds are included in different uses regulated under different legislative frameworks. In addition, one regulatory agency could take the lead in collecting the needs and stakeholder views on “cross-sectoral” case study proposals in the future.

#### 2.1.3. Introduce intermediate measures in line with precautionary approaches, e.g. Application of mixture assessment factors (MAFs)

Intermediate measures that could be implemented on a relatively short notice, include an additional assessment factor to account for mixture effects. This would be a way to decrease the total burden of exposure to chemical mixtures. It is evident that there is no single way to address all mixture exposure and toxicity issues in one simple approach; For empirical assessment e.g., the number of potential mixtures to test would be vast and such testing would not be feasible. Similarly, modelling based on simplistic assumptions would raise concerns of too many uncertain assumptions, so better ways of addressing both the scientific, regulatory as well as societal needs are necessary. Given the complexity of the problem, considerable uncertainties will remain for the coming decades. Thus, decisions about acceptable mixture exposures need to be made with some uncertainty, but taking benefit from stepwise translation of science.

Making better use of existing options that are included already in the current legislative frameworks, such as regulating substances as groups under REACH and making use of restrictions could be emphasized more when there is a concern of significant health or environmental impacts. The restriction proposal on the four phthalates, DEHP, BBP, DBP, and DIBP,^1^ was made on this basis (EC, 2018a).

An important question raised at the workshop was how to avoid a dilution of responsibilities: Even if every actor is responsible for just a small contribution to an overall intolerable exposure level, the question is how to get the overall exposure levels limited to “safe(r)” or acceptable levels. Some workshop participants were in favour of more rapid operationalization of intermediate measures as a way to decrease the total burden of involuntary exposure to chemical mixtures. Where the available evidence provides reasons for concern but a conclusive mixture risk assessment cannot be reached due to significant knowledge and data gaps and when potential serious and irreversible impacts are considered possible, intermediate measures, such as a mixture assessment factor (MAF), could be used to implement precautionary approaches.

- Application of mixture assessment factor (MAF)
  One concrete step forward could be the application of an additional assessment factor to account for possible combined exposures and effects. It has been proposed as a simple fixed factor for lower tier assessments to take account of mixture exposure in single substance risk assessment. The application of MAFs would furthermore increase the safety of the assessment of individual compounds. New research should develop realistic models of the relative contributions made by subgroups of chemicals to the total exposure, as this may help to further develop the MAF methodology. Some models are already available for informing about a possible range of estimating MAF, e.g. the acceptable concentration range models. These new statistical models deliver “guideline values” for risk based on epidemiological data to inform risk assessment of mixtures and provide estimates of acceptable exposures (Gennings et al., 2018).

#### 2.1.4. Integrate component-based and whole mixture testing approaches in common tiered frameworks for human and environmental mixture risk assessments

Existing proposals for tiered regulatory approaches to mixture risk assessment largely focus on component-based approaches (CBAs). These are scientifically reasonable but when translating them into regulation they are often limited in application, missing data on mixture components. Also, they may underestimate, or potentially overestimate, mixture risks that result from more/less than additive (synergistic/antagonistic) interactions. Where appropriate, complementary use of CBA and whole mixture testing approaches should be considered. This concept is currently under discussion at an advanced stage in the context of EU water-related legislation. In the Marine Strategy Framework Directive (MSFD, Directive 2008/56/EC), effect-based methods are included as supplementary descriptors to assess good environmental status on a voluntary basis. In environmental monitoring, comprehensive mixture exposure assessment is often limited by chemical analytic methodology. Here, joint application of effect-based methods and chemical analytics can help identify unresolved effects and compounds. Techniques and case studies for the proposed use of effect-based monitoring and related effect-based trigger values have been provided (Escher et al. 2018; Neale et al. 2017), as well as a concept of reference material mixtures based on main chemical drivers of the priority substances.

- Complementing target chemical monitoring with effect-based methods
  Strategies to complement chemical analytical data with effect detection have been proposed. They differentiate suggestions with regard to specific objectives, namely to identify relevant contaminants for complex exposure situations, to assess the impact of contamination in aquatic ecosystems, and to quantify cause-effect-relationships (Altenburger et al., 2019). For these different objectives they specify complementary applications of bioanalytical and chemical methods. Importantly, an array of effect-based methods is to be collated, e.g. for long-term effects of concern, such as endocrine activity that reflect the monitoring goal.

### 2.2. Key enablers for connecting the dots: systematic data and tools

#### 2.2.1. Complementary use of monitoring data and modelled exposure

Monitoring data can help identifying realistic co-exposure patterns if several chemicals are analysed e.g. in the same environmental or human samples. The use of Human Biomonitoring (HBM) data to identify realistic co-exposures and to help assess mixture related risks is gaining momentum. HBM data can account for combined exposure to multiple chemicals as well as aggregate exposure from different sources and via different routes, integrating, over time periods, bioaccumulating compounds. It was discussed that a complementation with modelling approach is warranted, e.g. predicting exposures based on chemical use types and volumes. Monitoring data can then be used to validate or calibrate such models. More generally, a full-scale computer-based strategy could be included in the planning phase of the initiative to increase interactions and exchanges between the continuous flow of big data from biomonitoring and exposure model outcomes. Such interactions also aim at improving exposure models. Practically, the development of the predictive models, which will rely on new data, can benefit from artificial intelligence techniques that use sophisticated algorithms. For example, machine learning techniques such as natural language processing, deep learning, random forest and Bayesian classifiers, to name a few, give the computer the ability to learn from data (here biomonitoring data) in order to make predictions. Such methods that have shown excellent performance in many fields, are fast and cost effective and should be helpful in many respects: (i) developing an integrated framework for cumulative and aggregated exposure, and improved predictive tools for internal doses based on exposure pathways; (ii) using physiologically-based toxicokinetic (PBTK) models to reconstruct exposures based on available information (reverse dosimetry); (iii) filling gaps in knowledge concerning environmental or human biomonitoring through adequate modelling; (iv) metabolic and kinetic interactions among mixture components; (v) identifying target tissue levels of contaminants and mixtures and therefore predicting the type of mixture present in target tissues; (vi) using large scale human biomonitoring data to identify co-occurring substances and mixture patterns in humans; this will also require specific tools based on combined data mining and statistical approaches to extract relevant features; (vii) based on available adverse outcome pathways (AOPs) and AOP networks, link relevant mixtures to adverse outcomes and predict toxicodynamic interactions; this could be done through innovative text mining and scoring tools combined to systems biology and using large scale data sets (omics).

- Additional and regular collection of human biomonitoring data
  Substitution of regulated or banned substances by often less well investigated “new” substances, shifts in the market, alterations in consumer habits, extended use of imported products from outside the EU and the introduction of new regulation require a continuous and regularly repeated screening of body burdens to elucidate real-world exposures and assess the major drivers in mixtures in people living in Europe, presently and at given future time points.

#### 2.2.2. Adequate and good-quality exposure and hazard data are prerequisites for MRA

Overall, the data availability and quality need to be improved. Predictive mixture assessment depends on adequate exposure and hazard data. Hazard and exposure data for mixture toxicity assessment are often lacking, scattered across disciplinary databases, or only cover a comparatively small number of chemicals, e.g. compounds considered relevant from a regulatory single-substance perspective or the “usual suspects” in academic (eco)toxicology and epidemiology.

- Coordination and data sharing within research community
  Many participants agreed that coordination efforts within the research community are needed to discuss ontologies, terminology and standardized formats and to make data findable with rich metadata. Such coordination could be possible to achieve via collaboration between funded projects, involvement of the Information Platform for Chemical Monitoring (IPCHEM), and relevant Commission services and agencies. Data should be stored in systematic, structured databases, with harmonised quality criteria. This would facilitate making data more accessible and comparable and help bridging the knowledge gaps. Likewise, international activities such as the AOP initiative of the OECD or US-EPA data integration efforts, e.g. Computational Toxicology Chemical Dashboard, could provide advanced data and knowledge sharing.

#### 2.2.3. Improving access to and transparency of data, including industry data

It was highlighted by several workshop participants that missing or inconsistent data on the toxicological properties of individual mixture components are a serious obstacle to component-based mixture risk assessment procedures. Information on the physico-chemical properties of chemicals, expected uses and related exposures, as well as toxicity data generated by industry for marketing approval or registration of chemicals, should be made transparently available. Also, as stated in the REACH Review (EC, 2018b), better quality of dossier information provided to authorities by industry is needed. Mechanisms or incentives to close such data and quality gaps, also across regulatory silos, are either missing or considered insufficient. To this end, appropriate enforcement and coordination measures must be implemented.

- Open access to data from industry studies
  As a concrete step forward, it was proposed that industry and business should be required to share data concerning exposure and application of chemicals, including life-cycle assessment data. In order to bring products to market, companies would have to test new substances regarding exposure and application. Further, information on chemicals added to materials and consumer products and goods should be made openly accessible. Large-scale users of chemical products with known active ingredient(s), such as farmers, would need to document their chemical product usage. These types of data are valuable and could contribute to risk assessment of chemicals and mixtures of chemicals. Therefore, legislation should require industry and users to share this data with authorities and make it publicly available. Also improved statistics on production, sale and amounts of chemicals used or applied would be essential to address data gaps or limitations in occurrence data.

### 2.3. Future research needs

#### 2.3.1. Coordinated research initiatives and research strategy

It is important to establish precise objectives, legislative and scientific, for the next ten years in the vast and complex field of chemical mixtures. Clear objectives could pave the road towards more consistent and complementary approaches and help avoid fragmentation, overlaps and gaps in research and efforts. Focused, coordinated, inter- and transdisciplinary research is needed that contributes to regulatory relevant issues but also towards wider understanding and new scientific knowledge.

#### 2.3.2. Perform targeted research into typical co-exposure patterns for humans and wildlife

In many situations, only few mixture components may dominate the overall risk. Further research on effect-based monitoring would help in validating modelled exposures and prioritising chemical mixtures for study. Risk reduction measures should consider such “drivers of toxicity”. However, the current knowledge on typical co-exposure patterns is only rudimentary and monitoring is focused on relatively few chemicals. Methods, such as untargeted screening, searching for emerging substances that populations are burdened with should already be included in approaches for monitoring. As we have limited ability to monitor all chemicals, the development of reliable and robust models is needed. These models have to be built on reliable and rich monitoring data, such as HBM data. Thus, large-scale research initiatives as well as relevant EU infrastructures for generating these data are required, and they should include both co-exposure modelling and monitoring. Further, there is a lack of information about the impact of chemicals on different species and their interaction with environmental factors. Eco-exposome research using large scale approaches, similar to some ideas developed in HBM4EU project, would be needed to study the presence of chemicals and their effects in the environment.

#### 2.3.3. Research and guidance for assessing risks from combined and sequential exposures

Progress has been made e.g. on dietary and non-dietary exposure models, but very few studies have been done on how these modelled exposures can be integrated in a single exposure estimate. Synergistic interactions that result in significant excess toxicity over expected additive effects are a long-standing concern of regulatory toxicology. The knowledge about such effects is fragmentary and episodic. Systematic investigations into the possible mechanisms and the possibilities to use such knowledge for predictive mixture risk assessments deserve support. Further, currently available methodologies for mixture risk assessment are largely confined to situations of simultaneous exposure to multiple toxicants. Sequential exposure plays an important role in real world scenarios but it is an under-researched issue. Research should focus on suitable concepts and method development for the predictive assessment of resulting risks.

#### 2.3.4. Strengthen the development of epidemiological approaches to mixture risk assessment

While traditional epidemiology has addressed the health effects of mixtures for decades through the use of exposure indicators, such indicators do not always allow for the breakdown of the mixtures into individual components. However, it can be difficult to disentangle the real-world exposures inherent in epidemiology studies and identify the drivers of observed health effects requiring remediation. Modern advances in epidemiological study designs, molecular epidemiology, exposome studies, epigenetics, statistical modelling and existence of large cohort studies enable more advanced use of epidemiology in mixture risk assessment. The development of these advanced epidemiological approaches to the assessment of mixture risks deserves further attention, as does the interaction with toxicological mixture risk research. The observational nature of epidemiological studies allows a better estimation of the possible magnitude of the mixture problem under real-life conditions.

#### 2.3.5. Develop further research based on the concepts of aggregate exposure pathway (AEP) and adverse outcome pathway (AOP)

Chemicals interact with one another and can result in effects that differ from the effects of the individual compounds. At this moment, there is no systematic method of investigating interaction other than by testing different combinations. The combination of AEP and AOP (Teeguarden et al., 2016; Escher et al., 2017) suggests that the interactions of chemicals can be organized into categories that may be more amenable to systematic research. To assess the mixture problem, organised knowledge is essential. These two frameworks, one well established, AOP, and the quite new AEP framework could help, in integrating mixture exposure information to link to health effect and disease and inform modelling and experimental studies.

#### 2.3.6. Integrated new approach methodologies (NAMs) for chemical testing

New approach methodologies (NAMs) addressing read-across and alternative methods to animal testing as defined and discussed elsewhere (ECHA, 2016) can provide quantitative mechanistic information on single substances and mixture combinations. Intensified research on NAMs will be essential to fill present knowledge gaps and overcome current limitations. Given the vast amount of possible mixture effects, such research should involve in silico approaches, advancing in vitro methodologies as well as high throughput methods using transcriptomics and apical end-points of toxicity testing. Such data will be critical to provide quantitative dose-response relation information on mixture effects for the scientific underpinning of mixture hazard identification and risk assessment. Integrated NAM testing will likely reduce uncertainties on the health effects of mixture exposures. Translation to epidemiological studies should involve refined biomarkers for adverse target organ effects.

#### 2.3.7. Transdisciplinary research on mixture risk governance

So far, little attention has been paid to the actual governance of chemical mixtures. A wide range of regulations apply to chemicals, and within the environmental, food safety and occupational arena, there is a growing recognition of the importance of chemical mixtures. The multitude and variability of exposure pathways makes the proper assessment of mixture exposures and assessments of mixture impacts complex and uncertain. Moreover, some of the exposures are involuntary (environmental, occupational, food), while others are largely self-determined (pharmaceuticals, alcohol, tobacco). Some have direct benefits to the individual, e.g. pharmaceuticals, while some do not, such as pesticides. This complexity leads to so-called ‘normative ambiguity’, e.g. where experts hold different value-based positions with regard to the acceptability of a risk, as opposed to ‘interpretative ambiguity’ where experts differ in the interpretation of the scientific evidence (Renn, 2008). This phenomenon also means that the complex, uncertain and ambiguous subject of mixture risk management cannot solely be executed through regulations, but it requires a wider involvement of multiple stakeholders. To our knowledge, so far such a transdisciplinary risk governance dialogue has not yet taken place. Pertinent questions to address in a transdisciplinary research agenda, for situations where the combined risks of mixtures are considered too high, would include:

- What rules would govern the risk reduction and who has the responsibility to reduce mixture risks?
- Should the main drivers of the risks be reduced, regardless of their exposure route and of individual or societal benefits?
- Should mixture risks in specific risk groups with the highest mixture exposures and risks be addressed first (“peak shaving”), or should the overall exposure distribution be reduced, to increase overall public health gain?
- How to ensure sufficient level of protection with regard to vulnerable groups, such as pregnant women and children?
- Should the individual and societal benefits play a role in optimizing approaches for mixture risk reduction, e.g. is there room for risk/benefit considerations?
- Should all exposure routes be equally reduced or involuntary exposures first?

Given the complexity of risk governance of mixtures and the unexplored territory of risk reduction schemes, a ‘sandbox’ approach of transdisciplinary research is proposed, to explore options and strategies that lead to efficient and effective mixture risk reduction, with sufficient societal support and minimum administrative burden.

#### 2.3.8. Progress to mixture risk-benefit assessment, including sustainability considerations

It is widely acknowledged that health and direct environmental risk are not the only relevant indicators for decision makers. In addition, more long-term sustainability factors and economic considerations are relevant in the decision-making process. Multi-criteria decision making (MCDM) models may be developed as part of governance research to address the various aspects of the complex mixture issue and provide further support for decision making.

## 3. Balancing uncertainties

Adding novel aspects to chemical risk assessment and surveillance is often met with scepticism based on conservative assumptions about uncertainty in other aspects of risk assessment and that additional considerations will be overly restrictive. This argument needs to be addressed in a comparative manner. Is there evidence for over-conservative assumptions regarding the various aspects of risk extrapolation, and can we aggregate factors for uncertainty across different sectors?

The complexity of mixture effects calls for a range of approaches to address all facets of the problem. Realistic mixtures can be derived from epidemiological and biomonitoring studies including untargeted screening and can then be tested. Relevant mixtures for study can also be identified through environmental monitoring or corresponding exposome studies. However, this requires collaboration and communication between the human health and ecosystems fields and the development of modelling approaches to link mixtures measured in the environment and human exposures.

The limitation of focusing exclusively on priority mixtures is that they may not provide a sufficiently comprehensive basis that would allow predictions to be made when new combinations are identified. In this respect, complementary component-based approaches, based on chemical groupings and mode of action, can be used to predict mixture effects. While a diversity of research directions is required, tools for regulatory decisions based on a tiered strategy should be rapidly implemented to support sufficiently protective decisions. These could be based on application of a MAF or use of default approaches such as dose addition. Such measures could be revisited with the development of additional knowledge.

Inescapably, there is a broad range of challenges to improve mixture risk assessment and management. Challenges include better understanding of mixture exposure patterns in populations and ecosystems; deeper insight into biology/mechanisms of adverse health and ecosystem effects; greater engagement with the social and policy/governance sciences; broader stakeholder dialogue with respect to possible avenues for mixture risk governance and about acceptability of regulatory measures. To initiate clear progress across these multiple challenges, extensive strategic transdisciplinary initiatives encompassing European and international collaborations are needed.

## Footnotes

1 Bis(2-ethylhexyl) phthalate (DEHP), Benzyl butyl phthalate (BBP), Dibutyl phthalate (DBP) and Diisobutyl phthalate (DIBP).

## References

[R1] Allen JH, 2013. The wicked problem of chemicals policy: opportunities for innovation. J. Environ. Stud. Sci 3, 101–108.

[R2] Altenburger R, Brack W, Burgess RM, Busch W, Escher BI, Focks A, Hewitt LM, Jacobsen BN, de Alda ML, Ait-Aissa S, Backhaus T, Ginebreda A, Hilscherova K, Hollender J, Hollert H, Neale PA, Schulze T, Schymanski EL, Teodorovic I, Tindall AJ, Umbuzeiro GD, Vrana B, Zonja B, Krauss M, 2019. Future water quality monitoring: improving the balance between exposure and toxicity assessments of real-world pollutant mixtures. Environ. Sci. Eur 31, 12.

[R3] Bopp SK, Barouki R, Brack W, Dalla Costa S, Dorne JCM, Drakvik PE, Faust M, Karjalainen TK, Kephalopoulos S, van Klaveren J, Kolossa-Gehring M, Kortenkamp A, Lebret E, Lettieri T, Norager S, Ruegg J, Tarazona JV, Trier X, van de Water B, van Gils J, Bergman A, 2018. Current EU research activities on combined exposure to multiple chemicals. Environ. Int 120, 544–562. 30170309 10.1016/j.envint.2018.07.037 PMC6192826

[R4] Bopp SK, Kienzler A, Richarz AN, van der Linden SC, Paini A, Parissis N, Worth AP, 2019. Regulatory assessment and risk management of chemical mixtures: challenges and ways forward. Crit. Rev. Toxicol 2019, 1–16. 10.1080/10408444.2019.1579169 30931677

[R5] Clahsen S.v.K., Hakkert B, Vermeire TH, Piersma AH, Lebret E, 2019. Why do countries regulate environmental health risks differently? A theoretical perspective. Risk Anal., vol. 39, 2, 439–461. 30110518 10.1111/risa.13165 PMC7379724

[R6] Demeneix B, Slama R, 2019. Endocrine disruptors: from Scientific Evidence to Human Health protection. Report for the Petitions Committee of the European Parliament. PE 608.866.

[R7] EC, European Commission, 2018a. COMMISSION STAFF WORKING DOCUMENT Accompanying the document COMMUNICATION FROM THE COMMISSION TO THE EUROPEAN PARLIAMENT, THE COUNCIL AND THE EUROPEAN ECONOMIC AND SOCIAL COMMITTEE Commission General Report on the operation of REACH and review of certain elements Conclusions and Actions SWD/2018/058 final, https://eur-lex.europa.eu/legal-content/EN/TXT/?uri=SWD:2018:58:FIN. 2018. (accessed 30 September 2019).

[R8] EC, European Commission, 2018b. Communication from the Commission to the European Parliament, the Council and the European Economic and Social Committee: Commission General Report on the operation of REACH and review of certain elements Conclusions and Actions. COM(2018)/0116 final (Accessed September 30, 2019).

[R9] ECHA, European Chemicals Agency, 2016. New Approach Methodologies in Regulatory Science. Proceedings of a scientific workshop Helsinki, 19-20 April 2016 <https://echa.europa.eu/documents/10162/21838212/scientific_ws_proceedings_en.pdf/a2087434-0407-4705-9057-95d9c2c2cc57> (Accessed September 30, 2019).

[R10] Escher BI, Hackermüller J, Polte T, Scholz S, Aigner A, Altenburger R, Böhme A, Bopp SK, Brack W, Busch W, Chadeau-Hyam M, Covaci A, Eisenträger A, Galligan JJ, Garcia-Reyero N, Hartung T, Hein M, Herberth G, Jahnke A, Kleinjans J, Klüver N, Krauss M, Lamoree M, Lehmann I, Luckenbach T, Miller GW, Müller A, Phillips DH, Reemtsma T, Rolle-Kampczyk U, Schüürmann G, Schwikowski B, Tan Y-M, Trump S, Walter-Rohde S, Wambaugh JF, 2017. From the exposome to mechanistic understanding of chemical-induced adverse effects. Environ. Int 99, 97–106. 27939949 10.1016/j.envint.2016.11.029 PMC6116522

[R11] Escher BI, Ait-Aissa S, Behnisch PA, Brack W, Brion F, Brouwer A, Buchinger S, Crawford SE, Du Pasquier D, Hamers T, Hettwer K, Hilscherova K, Hollert H, Kase R, Kienle C, Tindall AJ, Tuerk J, van der Oost R, Vermeirssen E, Neale PA, 2018. Effect-based trigger values for in vitro and in vivo bioassays performed on surface water extracts supporting the environmental quality standards (EQS) of the European Water Framework Directive. Sci. Total Environ 628–629, 748–765. 10.1016/j.scitotenv.2018.01.340 29454215

[R12] Evans RM, Martin OV, Faust M, Kortenkamp A, 2016. Should the scope of human mixture risk assessment span legislative/regulatory silos for chemicals? Sci. Total Environ 543, 757–764. 26573369 10.1016/j.scitotenv.2015.10.162

[R13] Gennings C, Shu H, Ruden C, Oberg M, Lindh C, Kiviranta H, Bornehag CG, 2018. Incorporating regulatory guideline values in analysis of epidemiology data. Environ. Int 120, 535–543. 30170308 10.1016/j.envint.2018.08.039 PMC6261378

[R14] Health Council of the Netherlands, 2008. Prudent precaution. Health Council of the Netherlands. The Hague. <https://www.sustainable-design.ie/arch/Prudent-Precaution_Netherlands-Health-Council_September-2008.pdf> (Accessed September 30, 2019).

[R15] Kienzler A, Berggren E, Bessems J, Bopp S, van der Linden S, Worth A, 2014. Assessment of mixtures - review of regulatory requirements and guidance. JRC Science for Policy Report, EUR 26675EN. Luxembourg: Publications Office of the European Union.

[R16] Kienzler A, Bopp SK, van der Linden S, Berggren E, Worth A, 2016. Regulatory assessment of chemical mixtures: requirements, current approaches and future perspectives. Regul. Toxicol. Pharm 80, 321–334. 10.1016/j.yrtph.2016.05.020 27211294

[R17] Kortenkamp A, Faust M, 2018. Regulate to reduce chemical mixture risk. Science 361, 224–226. 30026211 10.1126/science.aat9219

[R18] Lebret E, 2015. Integrated environmental health impact assessment for risk governance purposes; across what do we integrate? Int. J. Environ. Res. Public Health 13 (1). 10.3390/ijerph13010071 PMC4730462 26703709

[R19] Meek ME, Boobis AR, Crofton KM, Heinemeyer G, Van Raaij M, Vickers C, 2011. Risk assessment of combined exposure to multiple chemicals: a WHO/IPCS framework. Regul. Toxicol. Pharm 60, S1–S14. 10.1016/j.yrtph.2011.03.010 21466831

[R20] More SJ, Bampidis V, Benford D, Hougaard Bennekou S, Bragard C, Halldorsson TI, Hernandez-Jerez AF, Koutsoumanis K, Naegeli H, Schlatter JR, Silano V, Nielsen SS, Schrenk D, Turck D, Younes M, Benfenati E, Castle L, Cedergreen N, Hardy A, Laskowski R, Leblanc JC, Kortenkamp A, Ragas A, Posthuma A, Svendsen C, Solecki R, Testai E, Dujardin B, Kass G, Manini P, Jeddi MZ, Dorne J-L, Hogstrand C, 2019. Guidance on harmonised methodologies for human health, animal and ecological risk assessment of combined exposure to multiple chemicals. EFSA J. 17 (3), 5634. 10.2903/j.efsa.2019.5634 PMC7009070 32626259

[R21] Neale PA, Altenburger R, Ait-Aissa S, Brion F, Busch W, Umbuzeiro GD, Denison MS, Du Pasquier D, Hilscherova K, Hollert H, Morales DA, Novak J, Schlichting R, Seiler TB, Serra H, Shao Y, Tindall AJ, Tollefsen KE, Williams TD, Escher BI, 2017. Development of a bioanalytical test battery for water quality monitoring: fingerprinting identified micropollutants and their contribution to effects in surface water. Water Res. 123, 734–750. 28728110 10.1016/j.watres.2017.07.016

[R22] Petersen AC, van Asselt MBA, 2008. Conclusions and recommendations. In: Mathijssen J, Petersen AC, Besseling P, Rahman A, Don H (Eds.). Dealing with Uncertainty in Policymaking: The Hague/Bilthoven/Leiden: CPB/PBL/Rand Europe.

[R23] Renn O, 2008. Risk governance: Coping with uncertainty in a complex world. London.

[R24] Renn O, Graham P, 2005. White paper on risk governance towards an integrative approach. Geneva, Switzerland: International Risk Governance Council.

[R25] Rotter S, Beronius A, Boobis AR, Hanberg A, van Klaveren J, Luijten M, Machera K, Nikolopoulou D, van der Voet H, Zilliacus J, Solecki R, 2018. Overview on legislation and scientific approaches for risk assessment of combined exposure to multiple chemicals: the potential EuroMix contribution. Crit. Rev. Toxicol 48, 796–814. 30632445 10.1080/10408444.2018.1541964

[R26] Teeguarden J, Tan YM, Edwards S, Leonard J, Anderson K, Corley R, Kile M, Simonich S, Stone D, Tanguay R, Waters K, Harper S, Williams D, 2016. Completing the link between exposure science and toxicology for improved environmental health decision making: the aggregate exposure pathway framework. Environ. Sci. Technol 50 (9), 4579–4586. 26759916 10.1021/acs.est.5b05311 PMC4854780

